# Dr. William M. Hunter

**Published:** 1890-01

**Authors:** 


### Dr. William M. Hunter.

Died, in Cincinnati, Ohio, November 25th, 1889, William M. Hunter, in the seventieth year of his age. Dr. Hunter was born in the city of Louisville, Kentucky, December 25th, 1819. Early in life he removed to Philadelphia, Pa., where, after receiving a good English education, he took up the study of dentistry with Dr. Elijah Neal, then a prominent practitioner in that city,

In 1842 Dr. Hunter removed to Cincinnati, where he established and continued a very successful practice till 1864, when he removed to New York, where he entered into practice and remained till 1867, when he returned to Cincinnati and engaged in the practice of his chosen profession, which he pursued with his former energy and enthusiasm till advancing years and feeble health began to tell upon him, when he was compelled gradually to relinquish his practice which had hitherto so wholly absorbed his attention. In this state of affairs, however, he had the great gratification of knowing that he had a son who was able to take up and carry on the practice, as it by degrees passed from his own hands, which was undoubtedly a great comfort to him.

Dr. Hunter possessed an energy and an enthusiasm in his profession that was truly wonderful; he gave to it a devotion that was untiring, and that was the admiration of all who knew him. He was a man of most remarkable vitality, vigor and physical elasticity. He was rarely, if ever, disabled by sickness. He was of slight build, but every tissue of his body seemed to be so firmly knit together, that disease could make no inroads upon it. He met with an accident about five years before his death, in which he had a foot crushed by a car, which confined him to his room for several months. From this shock he never fully rallied, and to this is due, in a large measure, the exhaustion of his strength, resulting in his death.

He was married in 1844, in Philadelphia, to Miss Caroline Ashton. They had four children; two dying in infancy. Mrs. Hunter, Dr. F. A. Hunter, and Miss Clara Hunter reside in Cincinnati, the son well sustaining the enviable reputation established many years ago, and the daughter has won for herself a gratifying position as an educator.

In professional attainments and ability Dr. Hunter stood at the front, and he exercised a marked and extended influence with his professional confreres throughout this country. He was an excellent operator, and in prosthetic dentistry he had no superior, if an equal; his intellectual resources were great; his mind dwelt much on new devices and modes of practice, and his manipulative skill was such as to enable him to execute his highest mechanical conceptions. For many things now used, either improvements or new devices, the profession is largly indebted to Dr. Hunter.

He was so much occupied in the lines above indicated, that he wrote but little, but what he did in that direction, shows that had he given his efforts to the literature of his chosen calling, he would have been equally as successful as in other matters. He was a man of the strictest integrity and honor—one in whom his friends and the public generally placed the utmost confidence.

